# First Biologic Drug in the Treatment of RAS Wild-Type Metastatic Colorectal Cancer: Anti-EGFR or Bevacizumab? Results From a Meta-Analysis

**DOI:** 10.3389/fphar.2018.00441

**Published:** 2018-05-03

**Authors:** Alessandro Ottaiano, Alfonso De Stefano, Monica Capozzi, Anna Nappi, Chiara De Divitiis, Carmela Romano, Lucrezia Silvestro, Antonino Cassata, Rossana Casaretti, Salvatore Tafuto, Michele Caraglia, Massimiliano Berretta, Guglielmo Nasti, Antonio Avallone

**Affiliations:** ^1^Department of Abdominal Oncology, Istituto Nazionale Tumori di Napoli “G. Pascale” IRCCS, National Cancer Institute, Naples, Italy; ^2^Department of Biochemistry, Biophysics and General Pathology, University of Campania “L. Vanvitelli” of Naples, Naples, Italy; ^3^Department of Medical Oncology, CRO Aviano, National Cancer Institute, Aviano, Italy

**Keywords:** colorectal carcinoma, chemotherapy, cetuximab, panitumumab, bevacizumab

## Abstract

**Introduction:** We performed a meta-analysis in order to analyze and quantify the effect on survival of starting therapy in RAS wild-type (wt) metastatic colorectal cancer (mCRC) patients with anti-EGFR agents or bevacizumab.

**Patients and Methods:** Randomized, phase II or III, clinical trials reporting overall survival (OS) in RAS wt mCRC patients treated with first-line chemotherapy (CT) associated with bevacizumab or anti-EGFR agents were selected. The primary end-point of this meta-analysis was OS; findings were depicted in classical Forest plots.

**Results:** Seven studies met the criteria for meta-analysis including 3,805 patients. The pooled second-line cross-over rate to bevacizumab was 36.6%, to anti-EGFR 33.2%. Only one study was selected reporting comparison between CT vs. CT plus bevacizumab in RAS wt patients with a HR of 1.13 in favor of CT (CI: 0.89–1.43, *p* = 0.317). The pooled HRs were 0.89 (95% CI: 0.79–1.00) for CT plus anti-EGFR vs. CT and 0.81 (95% CI: 0.71–0.92) in favor of CT plus anti-EGFR vs. CT plus bevacizumab. Subgroup analysis showed a positive prognostic impact of starting CT plus anti-EGFR in left colon cancer (pooled HR: 0.70; CI: 0.54–0.85) while a positive trend of starting CT plus bevacizumab was observed in right colon cancer (pooled HR: 1.29; CI: 0.81–1.77).

**Conclusions:** This meta-analysis shows that starting therapy in RAS wt mCRC patients with an anti-EGFR agent improves OS when the primary tumor location is in the left colon but a strong limitation of previous studies is the very low rate of biologic drug therapy cross-over.

## Introduction

Metastatic colorectal cancer (mCRC) is the third leading cause of cancer-related deaths worldwide (Siegel et al., 2017). About 20 percent of patients with colorectal cancers have clinical evidence of metastases at diagnosis (Fiorentini et al., 2017). Unfortunately, less than 10% of patients can undergo to surgical removal of metastases, the large part of mCRC patients are candidate to first-line chemotherapy. Over the last 15 years, new drugs, both cytotoxic (fluoropyrimidines, oxaliplatin, irinotecan) and biologic agents (cetuximab, panitumumab, bevacizumab), have determined a significant improvement of both objective response rate and overall survival (OS) in the first-line treatment of mCRC patients (Nappi et al., 2017).

Anti-EGFR agents are represented by cetuximab (chimeric mouse-human mAb IgG1) and panitumumab (humanized mAb IgG2): they bind to EGFR (Epidermal Growth Factor Receptors) preventing the activation of downstream signal proteins and the proliferative effect of its natural ligands in neoplastic cells. Another effect of cetuximab is also the activation of immune response through antibody-dependent cell cytotoxicity (ADCC). Clinical trials demonstrated the efficacy of anti-EGFR agents both in association with doublets (FOLFIRI and FOLFOX) and monotherapy (De Divitiis et al., 2014; Nappi et al., 2016, 2017). Many studies demonstrated the predictive role of RAS mutational status for the efficacy of anti-EGFR agents (Lo Nigro et al., 2016), thus the use of these agents is recommended by the National Comprehensive Cancer Network, European Society for Medical Oncology, and Japanese Society for Cancer of the Colon and Rectum, only in mCRC patients with wild-type KRAS and NRAS (“RAS status”). However, the prediction of response to first-line anti-EGFR therapy is a dynamic issue and several other gene alterations are candidate biomarkers for predicting the efficacy of anti-EGFR treatment (De Roock et al., 2010; Seo et al., 2014) as well as toxicity to treatments (Catapano et al., 2014).

Bevacizumab is a humanized monoclonal IgG1 antibody targeting the vascular endothelial growth factor (VEGF); therefore, it prevents the binding to the VEGF receptor exhibiting many different anti-angiogenic and anti-tumor effects. Clinical studies have demonstrated that chemotherapy doublets (FOLFIRI or FOLFOX) in association with bevacizumab are active independently from RAS status. Nowadays, bevacizumab is indicated in association with chemotherapy (CT) in 1st and 2nd treatment lines of mCRC patients and no biomarkers are known in order to predict response or resistance to it (Van Cutsem et al., 2016).

The introduction of new drugs has improved median survival of mCRC from 12 months with fluorouracil monotherapy to more than 24 months. However, data on different therapy sequences are still controversial, particularly in RAS wild-type (wt) mCRC patients. It has been shown that the best survival is reached when all drugs are administered in a context of a continuum of care (Goldberg et al., 2007). This is a clinical paradigm apparently in contrast with the concept of different schedule sequences as a possible therapeutic strategy.

Since intensive debate is about which biologic drug should be associated with CT as front-line treatment of RAS wt mCRC we performed a meta-analysis including the most recent published data and the most updated time-to-outcome results; additionally, a systematic and detailed report of subsequent lines of therapy in RAS wt metastatic patients is also reported.

## Patients and methods

### Design and trial identification criteria

**Figure 1:**
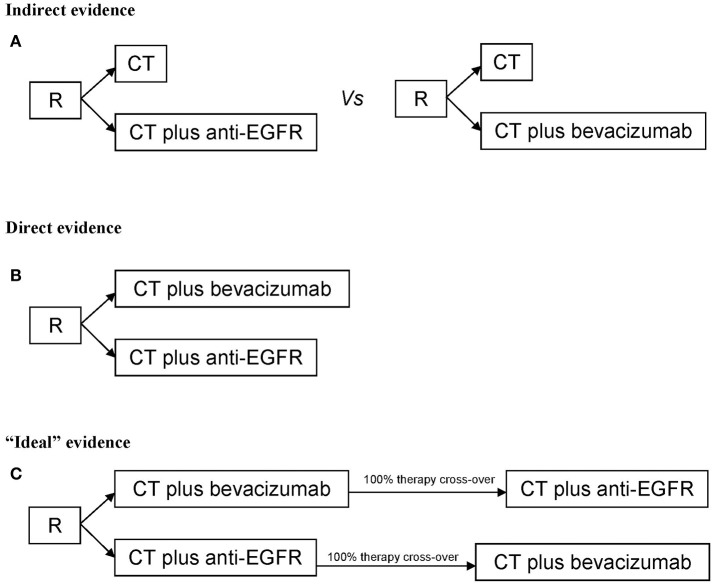
Study design for **(A)** Indirect evidence, **(B)** direct evidence, **(C)** “ideal” evidence for quantifying the extent of benefit on survival of starting therapy in metastatic colorectal cancer patients with anti-EGFR agents or bevacizumab. R, randomization; CT, chemotherapy.

The present meta-analysis was performed to analyze and quantify the effect on OS of starting therapy of RAS wt mCRC with anti-EGFR agents or bevacizumab in combination with CT. Two types of studies were selected: the first consists on randomized studies reporting on CT plus different biologic drugs vs. CT only (where CT only works as normalization arm for inter-trials comparisons) and it provides an indirect and exploratory evidence; the second consists on the comparisons/randomization between different biologic drugs (CT plus A vs. CT plus B) and it provides a direct evidence (Figure 1). Randomized, phase II or III, first-line clinical trials reporting OS as primary or secondary outcome in RAS wt patients or K-RAS when the extended mutational status was not available, published in the last ten years were eligible. Only papers published in peer-reviewed journals and in English were considered. Ancillary studies, reviews, opinions, meta-analyses, maintenance studies with an induction phase less than 6 months were also excluded. When more publications regarding the same clinical trial were present, the work reporting the most updated survival was used for meta-analysis; if necessary, clinical and pathological characteristics were derived from previous reports.

### Search strategy

**Figure 2:**
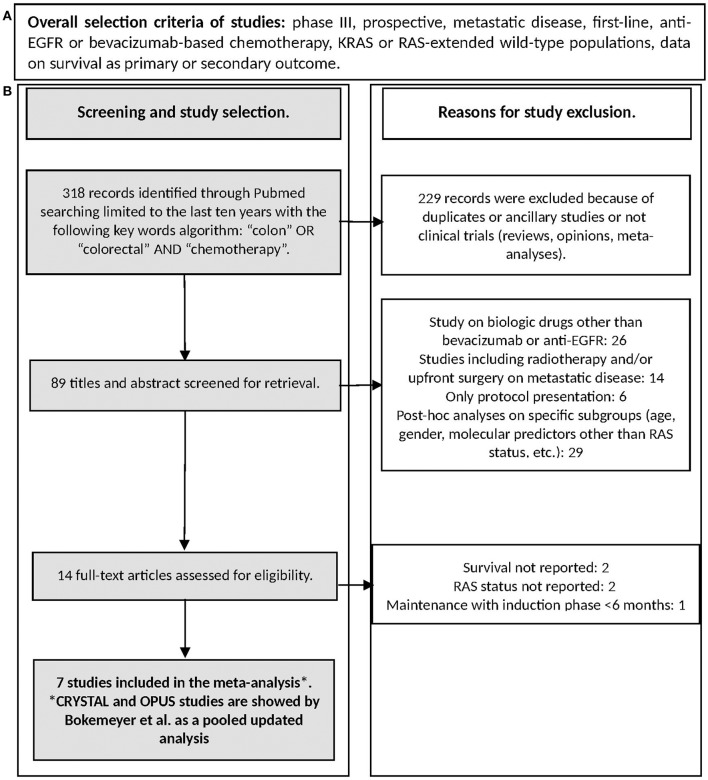
**(A)** Citreia for study selection, **(B)** Study selection flow chart.

Search was performed on July 2017 through Medline (PubMed: www.ncbi.nlm.nih.gov/ PubMed) and the registry of the US National Institutes of Health clinicaltrials.gov. Keywords used for searching were “colon,” “colorectal,” “chemotherapy” limiting the research to clinical trials. References of selected articles were also checked in order to find additional reports. The flow-chart reporting the search strategy (study selection and exclusion) is shown in Figure 2.

### Data extraction

The following data were extracted for each publication: first author; year of publication; arms; phase; number of randomized patients according to KRAS o “RAS extended” status; information on subsequent biologic therapy (biologic drug cross-over); accrual time; median survival with CI; hazard ratios with CI; *P* at log-rank test; age; gender; PS ECOG; overall response rate. All data, particularly the time-to-outcome, were extracted in RAS wt population and were reviewed and separately computed by two investigators (M.C. and A.O.). Criticisms and discordances were discussed with all authors.

### Study quality

The Method for Evaluating Research and Guideline Evidence (MERGE) criteria (Liddle et al., 1996) were applied to assess quality of studies. All studies had an overall quality score of A (low risk of bias) or B1 (low to moderate risk of bias) (data not shown).

### Statistical methods

A meta-analysis was performed in order to evaluate the overall effect on OS of starting therapy with different biologic therapies in association with CT in mCRC patients. Thus, the primary end-point of this meta-analysis was overall survival, defined as the time between date of random assignment and date of death, or last date of follow-up for censored patients. The description of the objective response rate was a secondary end-point. The results were extracted as hazard ratios (HRs) of OS. The meta-analysis was done with the fixed-effect model assuming that the studies which have the same effect or meaning were homogeneous. The analysis was also performed with the random-effect model taking in account the alternative hypothesis of heterogeneity. The combined analysis included the Cochrane's Q-test for the heterogeneity in the studies. Sub-group meta-analyses were performed if at least two studies were available. To allow HRs comparisons, findings of the meta-analysis are also depicted in classical Forest plots, with point estimates and 95% CIs for each trial and overall; size of the squares is proportional to study size. Publication bias was evaluated by the construction of funnel plot (Egger et al., 1997) where any asymmetry of the graph indicate some sort of heterogeneity (statistical, methodological, or clinic) and/or publication bias. Analyses were performed with the MedCalc Statistical Software version 16.2.1 (MedCalc Software bvba, Ostend, Belgium; https://www.medcalc.org; 2016).

## Results

### Characteristics of studies

Seven studies met the criteria for meta-analysis (CT vs. CT plus biologic therapy or direct comparison between CT plus anti-EGFR vs. CT plus bevacizumab) (Maughan et al., 2011; Bokemeyer et al., 2012; Douillard et al., 2013; Schwartzberg et al., 2014; Passardi et al., 2015; Stintzing et al., 2016; Venook et al., 2017) including 3,805 patients KRAS or “RAS extended” wt. However, only one study comparing CT vs. CT plus bevacizumab was selected (Passardi et al., 2015), thus this study was not included in a meta-analysis model; HR and CI are shown in the Forest Plot to allow a descriptive comparison. Characteristics of selected studies are reported in Table 1. The number of RAS selected patients included in each trial ranged from 170 to 730. Information on second line therapies is not reported in three studies. The pooled cross-over rate to bevacizumab (first anti-EGFR then bevacizumab) is 36.6%, to anti-EGFR (first bevacizumab then anti-EGFR) is 33.2%. The accrual time ranged from 20 to 90 months. Blinding was never present. Median survival for patients treated with biologic therapies ranged from 19.9 to 41.3 months. HRs for survival with CI are also reported; only two studies (Bokemeyer et al., 2012; Stintzing et al., 2016) reported a statistically significant survival gain (CT vs. CT plus cetuximab: +3.7 months in favor of CT plus cetuximab; CT plus cetuximab vs. CT plus bevacizumab: +8.1 months in favor of CT plus cetuximab).

**Table 1:** Characteristics of randomized trials included in the meta-analysis.

**Author and trial**	**Year**	**Arms**	**Phase**	**Randomization according to RAS wild-type**	**Information on subsequent lines of therapy: bevacizumab (B) or anti-EGFR-based (E)**	**Start accrual-End accrual**	**Median survival (months)**	**No. of events**	**CI**	**Hazard ratio**	**CI**	***P***
**CT VS. CT PLUS ANTI-EGFR**												
*Maughan TS*	2011	CT	III	289	E: 16; B: NR	March 2005–May 2008	20.1	156	11.5–31.7	1.02	0.83–1.24	0.86
MRC COIN		CT/Cet		292	E: 21; B: NR		19.9	156	For all pts RAS wt			
*Bokemeyer C*	2012	CT	III	381	NR	July 2004–March 2006	21.1	295	19.5–23.6			
CRYSTAL and OPUS		CT/Cet		349	NR		24.8	255	22.1–27.0	0.84	0.71–1.00	0.048
*Douillard JY*	2013	CT	III	253	E: 25.4% B:NR	Not reported	20.2	218	17.6–23.6			
PRIME		CT/Pan		259	E: 12.9% B: NR	Not reported	25.8	204	21.7–29.7	0.77	0.64–0.94	0.009
**CT vs. CT PLUS BEVACIZUMAB**												
*Passardi A*	2015	CT	III	98^A^	NR	November 2007–March 2012	21.3	89	19.9–24.1			
ITACa		CT/Beva		91^A^	NR		20.8	85	15.9–23.2	1.13	0.89–1.43	0.317
**CT PLUS ANTI-EGFR vs. CT PLUS BEVACIZUMAB**												
*Schwartzberg LS*	2014	CT/Pan	II	88	B: 35	Apr 2009–Dec 2011	41.3	50	28.8–41.3			
PEAK		CT/Beva		82	E: 30		28.9	60	23.9–31.3	0.63	0.39–1.02	0.058
*Stintzing S*	2016	CT/Cet	III	199	B: 60	Jan 2007–Sept 2012	33.1	107	24.5–39.4			
FIRE-3		CT/Beva		201	E: 54		25.0	133	23.0–28.1	0.70	0.54–0.90	0.0059
*Venook AP*	2017	CT/Beva	III	559^A^	NR	Sept 2005–March 2012	30.0	242	NR			
CALGB/SWOG 80405		CT/Cet		578^A^	NR		29.0	240	NR	0.88	0.77–1.01	0.08

A *Information is available only for KRAS. CT, chemotherapy; Cet, Cetuximab; WT, wild-type; Pan, Panitumumab; Beva, Bevacizumab; CI, confidence interval; NR, Not reported. Data are reported only in KRAS (KRAS wt) or in “RAS extended” wild-type population (KRAS and NRAS wt)*.

### Characteristics of patients

Age, gender, and PS ECOG of enrolled patients are described in Table 2. The median ages ranged from 59 to 65 years. The enrollment of male patients was predominant and this reflects the epidemiology of colorectal cancer. The most prevalent PS according to the ECOG scale were 0 and 1. COIN, CRYSTAL, OPUS, and PRIME studies enrolled also a significant percentage of PS ECOG 2 or 3 patients; those patients were homogeneously distributed between arms so that results were unlikely to be biased. However, inter-trial results could be influenced by PS of patients which is one of the most important prognostic factor in colorectal cancer.

**Table 2:** Characteristics of patients included in the meta-analysis.

**Trial**	**Arms**	**Age**	**Gender**		**PS ECOG**			
		**median (range)**	**Male**	**Female**	**0**	**1**	**2**	**3**
MRC COIN	CT	63 (56–69)	245	122	177	166	24	0
	CT/Cet	64 (59–70)	253	109	171	171	20	0
					**0/1**			
CRYSTAL and OPUS	CT	59 (19–84)	228	153	363		18	0
	CT/Cet	61 (24–79)	214	135	332		17	0
					**0/1**		**2/3**	
PRIME	CT	61 (24–82)	204	126	312		18	
	CT/Pan	62 (27–85)	217	108	305		20	
						**1/2**		
ITACa^A^	CT	66 (32–82)	115	79	154	40	0	0
	CT/Beva	66 (34–83)	108	68	144	32	0	0
FIRE-3	CT/Cet	64 (41–76)	145	60	98	102	5	0
	CT/Beva	65 (33–76)	133	69	105	94	3	0
PEAK	CT/Pan	62 (23–82)	58	30	53	35	0	0
	CT/Beva	60 (39–82)	56	26	52	29	0	0
CALGB/SWOG 80405^A^	CT/Beva	59.0 (21.8–85.0)	348	211	324	233	2	0
	CT/Cet	59.2 (20.8–89.5)	349	229	333	245	0	0

A *Information is not available in RAS selected but in all patients. PS, Performance Status; ECOG, Eastern Cooperative Oncology Group; CT, chemotherapy; Cet, Cetuximab; Pan, Panitumumab; Beva, Bevacizumab. In some cases the sum does not correspond to the enrolled patients because of differences between “RAS assessable” and RAS evaluated patients*.

### Overall response and resection rates

Response rates are described in Table 3 and ranged from 25.6% (CT plus bevacizumab) to 64.0% (CT plus cetuximab). The percentage or the number of patients in each trial who received metastasectomies are also shown; they were homogeneously low (always <20%).

**Table 3:** Overall response and resection rates of studies included in the meta-analysis; if not specified, data refer to KRAS or RAS “extended” wt patients.

		**Overall response rate**				**Patients underwent to metastasectomies^B^**	
**Trial**	**Arms**	**CR No or %**	**PR No or %**	**SD No or %**	**PD No or %**	**%**	**No**
MRC COIN	CT	209		NR	NR		12
	CT/Cet	232		NR	NR		13
		**CR/PR**					
CRYSTAL and OPUS	CT	40.9%		NR	NR	Crystal: CT 3.7% vs. CT/Cet 7.0%	
	CT/Cet	60.7%		NR	NR	OPUS: CT 4.1% vs. CT/Cet 9.8%	
		**CR/PR**					
PRIME	CT	154		NR	NR	8.0	
	CT/Pan	181		NR	NR	10.0	
		**CR/PR**		**SD/PD**			
ITACa	CT	26.8%		73.2%		18.7^A^	
	CT/Beva	25.6%		74.4%		18.6^A^	
PEAK	CT/Pan	2	54	23	1	13	
	CT/Beva	1	48	22	4	11	
FIRE-3	CT/Cet	9	103	53	10		36^A^
	CT/Beva	2	100	85	8		40^A^
		**CR/PR**					
CALGB/SWOG 80405	CT/Beva	55.2%^A^		NR	NR	Twelve percent of patients were	
	CT/Cet	59.6%^A^		NR	NR	disease free with metastasectomies^A^.	

*Data are reported on the ITT populations. In some cases the sum of response rate do not result 100% because of unevaluable cases. CT, chemotherapy; Cet, Cetuximab; Pan, Panitumumab, Beva, Bevacizumab. CR, complete response; PR, partial response; SD, stable disease; PD, progressive disease; NR, not reported*.

A Data refer to all patients;

B *This data does not imply R0 resection*.

### Overall survival according to first-line biologic drug

**Figure 3:**
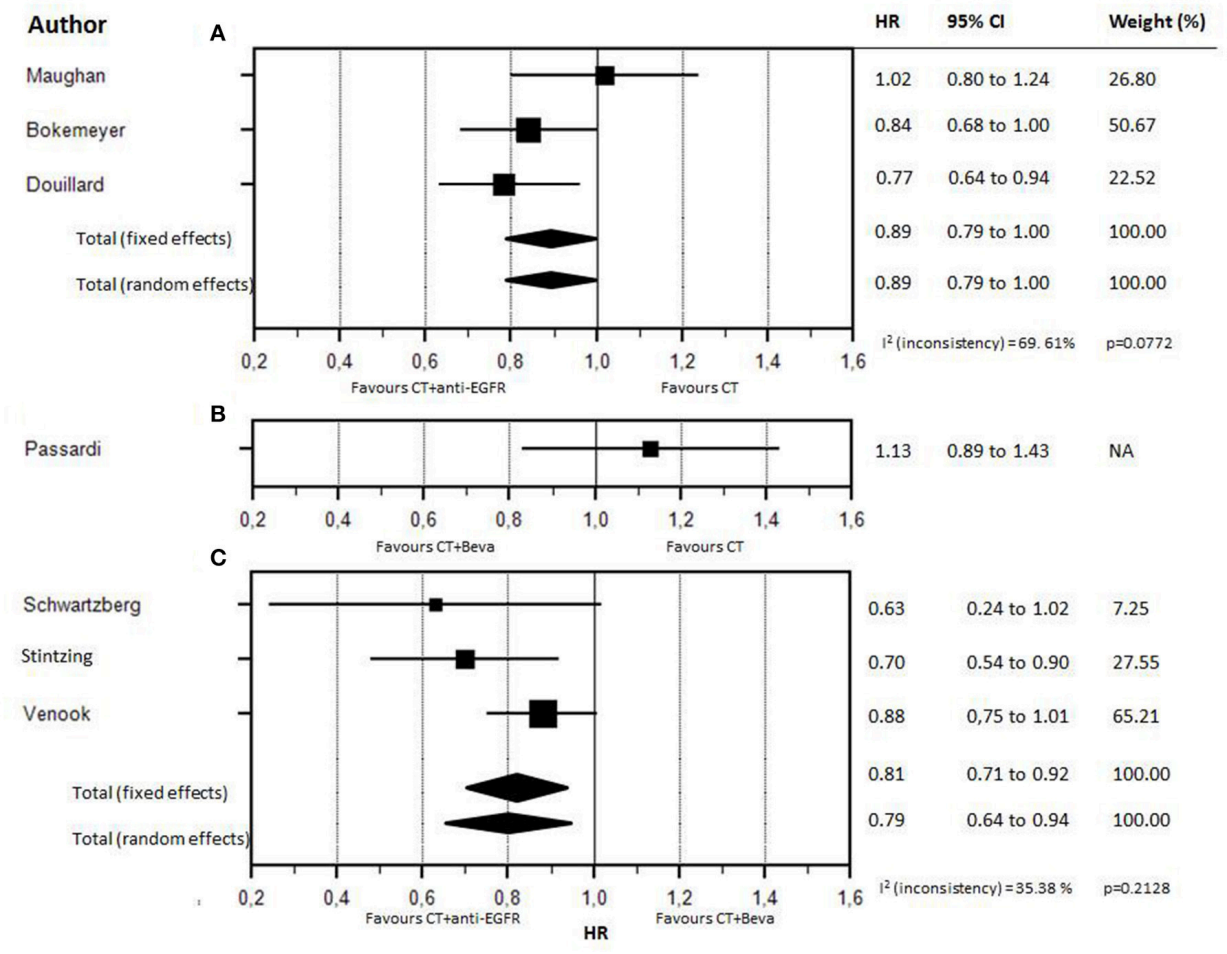
Forest plot for OS according to different firs-line biologic drugs. Forest plot for OS according to different first-line biologic drugs. **(A)** CT (chemotherapy) vs CT plus anti-EGFR. **(B)** CT vs CT plus bevacizumab. **(C)** CT plus anti-EGFR vs CT plus bevacizumab.

**Figure 4:**
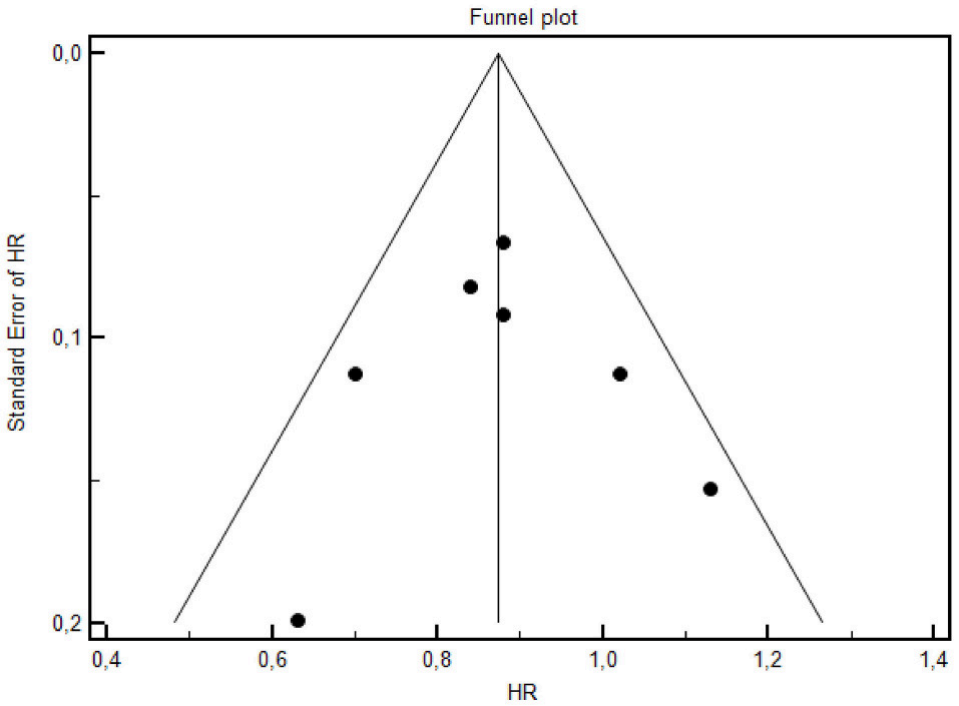
The funnel plot for OS of selected studies.

Forest plot of treatments' effect on OS is shown in Figure 3. There was no statistically significant heterogeneity among the seven trials (*P* = 0.77). The HR of the pooled subgroup meta-analysis were 0.89 (95% CI: 0.79–1.00) in favor of CT plus anti-EGFR vs. CT and 0.81 (95% CI: 0.71–0.92) in favor of CT plus anti-EGFR vs. CT plus bevacizumab. Funnel plot did not show publication bias (Figure 4). HR of Passardi et al. (CT vs. CT plus bevacizumab) is descriptively shown in the Forest Plot (1.13, CI: 0.89–1.43, in favor of CT, *p* = 0.317).

### Overall survival according to primary tumor location

**Figure 5:**
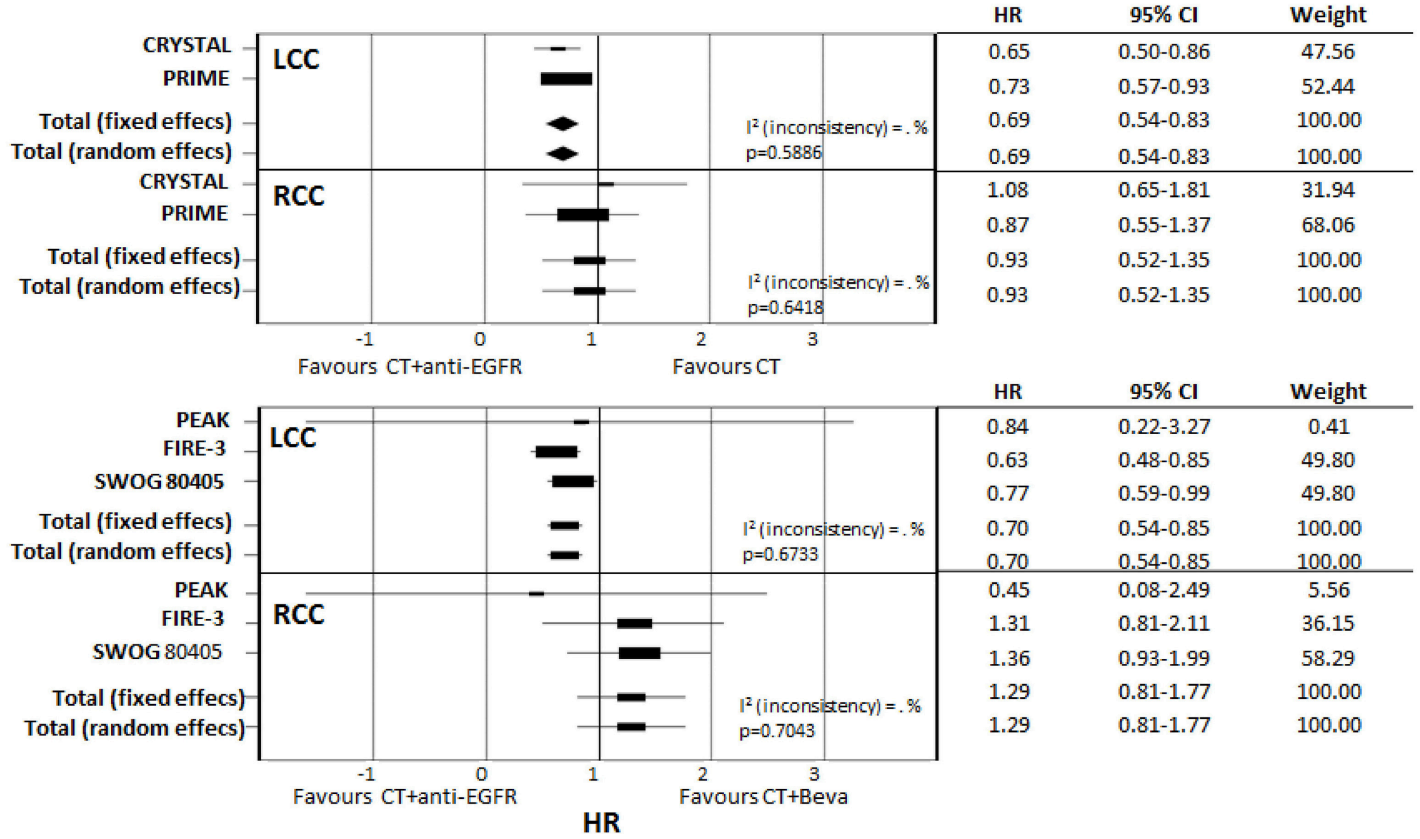
Subgroup meta-analysis of primary tumor location effect on survival according to different first-line biologic drugs.

Detailed data on the impact of primary tumor location on survival in RAS wt patients were available in five studies (Table 4): CRYSTAL (Tejpar et al., 2016), PRIME (Douillard et al., 2013), PEAK (Schwartzberg et al., 2014), FIRE-3 (Stintzing et al., 2016), and SWOG 80405 (Venook et al., 2017). Meta-analysis of CRYSTAL and PRIME shows that starting CT with anti-EGFR was favorable in LCC compared with CT only (pooled HR: 0.69; CI: 0.54–0.83); by contrast, in RCC there was no significant difference when adding an anti-EGFR in first-line therapy (pooled HR: 0.93; CI: 0.52–1.35). Meta-analysis of studies reporting on direct comparisons of biologic therapies (CT plus anti-EGFR vs. CT plus bevacizumab) showed a clear positive prognostic impact of starting CT plus anti-EGFR in LCC (pooled HR: 0.70; CI: 0.54–0.85) while a positive trend in favor of starting CT plus bevacizumab was observed in RCC (pooled HR: 1.29; CI: 0.81–1.77) (Figure 5).

**Table 4:** Effect of primary tumor site on survival of RAS wt mCRC patients treated with different biologic drugs.

**Trial**	**Primary tumor site**	**Arms**	**No. of patients**	**No. of events**	**Median survival**	**Hazard Ratio**	**CI**	***P***
CRYSTAL	LCC	CT	138	112	21.7	0.65	0.50–0.86	0.02
		CT/Cet	142	102	28.7			
	RCC	CT	51	42	15.0	1.08	0.65–1.81	0.76
		CT/Cet	33	26	18.5			
PRIME	LCC	CT	159	136	23.6	0.73	0.57–0.93	NR
		CT/Pan	169	126	30.3			
	RCC	CT	49	44	15.4	0.87	0.55–1.37	NR
		CT/Pan	39	34	11.1			
PEAK	LCC	CT/Pan	53	29	43.4	0.84	0.22–3.27	NR
		CT/Beva	54	33	32.0			
	RCC	CT/Pan	22	19	17.5	0.45	0.08–2.49	NR
		CT/Beva	14	12	21.0			
FIRE-3	LCC	CT/Beva	149	106	28.0	0.63	0.48–0.85	0.002
		CT/Cet	157	86	38.3			
	RCC	CT/Beva	50	38	23.0	1.31	0.81–2.11	0.28
		CT/Cet	38	31	18.3			
CALGB/SWOG 80405	LCC	CT/Beva	152	119	32.6	0.77	0.59–0.99	0.04
		CT/Cet	173	119	39.3			
	RCC	CT/Beva	78	58	29.2	1.36	0.93–1.99	0.10
		CT/Cet	71	56	13.7			

*LCC, left colon cancer; RCC, right colon cancer; CT, chemotherapy; Cet, Cetuximab; Pan, Panitumumab; Beva, Bevacizumab; CI, confidence interval; NR, Not Reported*.

## Discussion

The actual therapeutic paradigm of mCRC treatment is that the exposure to all active drugs may reach the highest survival. This is apparently in contrast with the concept of therapy sequences. The important question on which regimen to use and in what sequence to administer is highly debated and it has gained much more importance particularly after the introduction of biologic drugs (cetuximab, panitumumab, bevacizumab) and the selection of patients on the basis of RAS status. In fact, it has been proposed that the tumor biology and the response to treatments may be influenced by different sequential biologic therapies. Meta-analysis is the best tool to give evidence-based answers to some clinical situations; the present meta-analysis shows that starting therapy in RAS wt mCRC patients with anti-EGFR improves OS particularly in LCC mCRC patients. Interestingly, specific findings about the differential role of primary tumor location are consistent with recent meta-analyses where the primary end-point was the prognostic or predictive effect of primary tumor side (Arnold et al., 2017; Holch et al., 2017); their end-points and design were different from the present one that shows the most specific and updated prognostic information in RAS wt mCRC patients. In particular, we selected two types of studies in order to quantify the extent of benefit obtained with different biologic drugs in first line therapy: (i) randomized studies reporting on CT plus biologic drug vs. CT only where CT only works as normalization arm for inter-trials comparisons; (ii) randomized studies reporting on direct comparisons between different biologic drugs. Meta-analysis of the first type of evidence gives an exploratory estimate of efficacy differences. Thus, our study cannot be considered as a subgroup of previous meta-analyses.

With regard to the use of bevacizumab in 1st line therapy, only one study comparing CT vs. CT plus bevacizumab met the criteria for inclusion (Passardi et al., 2015); two other studies in literature (Cremolini et al. TRIBE, Hegewisch-Becker et al. AIO0207) (Cremolini et al., 2015; Hegewisch-Becker et al., 2015) reported data on CT plus bevacizumab as first-line therapy in RAS wt patients but there was not a formal control arm with chemotherapy only (CT) or chemotherapy with anti-EGFR; therefore, they were excluded. However, median survival of these studies compared well with the widely described survival of last generation trials (about 30 months). Notably, in the study by Cremolini et al. (2015) the cross-over rate to anti-EGFR was 21.8%, in the study by Hegewisch-Becker et al. (2015) was 68.3%. Heterogeneity in rates of antibody crossover is a clinical and methodological issue that may influence the final outcome, particularly OS which is influenced by all lines of therapy.

In studies reporting on the direct comparison of sequences (Schwartzberg et al., 2014; Stintzing et al., 2016; Venook et al., 2017), results of meta-analysis are in favor of starting with anti-EGFR agents (pooled HR, fixed model 0.81, CI: 0.71–0.92; pooled HR, random model 0.79, CI: 0.64–0.94). In last years, many data have showed that tumors arising in different sites of the colon are different in terms of embryonic derivation, molecular alterations, and clinical prognosis (Maus et al., 2015; Tejpar et al., 2016); two main groups can be identified: (i) left colon cancers (LCC) that comprise the distal one-third of transverse colon, splenic flexure, descending and sigmoid colon, rectum, and (ii) right colon cancers (RCC) including appendix, cecum, ascending colon, hepatic flexure, and two-thirds of proximal transverse colon. The advantage of starting chemotherapy with anti-EGFR agents was evident only in LCC (pooled HR: 0.70; CI: 0.54–0.85) but also these results may be influenced by the low rate of patients who received bevacizumab in further lines of chemotherapy. PS, age and gender were well-balanced between trials as well as the quality of studies (according to MERGE criteria).

The ideal study design to analyze the role of different biologic drugs in 1st line therapy consists on a preplanned high cross-over rate of biologic therapies (true comparison of different sequences therapy) (Figure 1). Thus, a limitation of the published studies is the very low rate of cross-over to the different biologic drugs. Biologic therapies are unexplainably underused in additional lines of treatment; when this data was reported, only 36.6% of patients starting with anti-EGFR were treated in further lines of therapy with bevacizumab, and only 33.2% of patients with RAS wt received an anti-EGFR agent. Thus, we cannot rule out the hypothesis that the negative effect of starting CT plus anti-EGFR in RCC could be regained by a higher therapeutic cross-over to bevacizumab.

Results of therapy in RCC mCRC patients can be also conditioned by numerous clinical and pathological factors. In fact, it was recently reported that RCC has larger tumor size, poor differentiation, advanced TNM stage, and shorter survival and this can influence the response to the administered therapy (Maus et al., 2015). It was also reported in some studies higher incidence of BRAF mutations in RCC that could reduce the efficacy of anti-EGFR agents (Gao et al., 2017). For this reason, the BRAF status should be an additional stratifying factor in therapeutic as well as prognostic studies in CRC patients. Indeed, Uivi et al. reported in a series of 370 patients that baseline inflammatory indexes were significantly higher in LCC, whereas eNOS and EPHB4 expression were significantly higher and BRAF mutations more frequent in RCC (Ulivi et al., 2017). In another study enrolling 1,319 patients a higher incidence of BRAF mutation was also found in RCC (RCC 26.6%, LCC 3.2%, *p* < 0.001) (Nitsche et al., 2016). Thus, it would be interesting to know at least the mutational status of BRAF according to primary tumor location in the surgical samples of the patients enrolled in the clinical trials included in the present meta-analysis in order to interpret the results on the different outcome reached in RCC and LCC. However, it is also like that the deep genetic analysis of the tumors through next generation sequencing could give more information on this in the future discovering new genetic biomarkers predictive of response to different therapy sequences in mCRC patinets. Furthermore, in the studies by Venook et al. and Passardi et al. patients selection was based only on KRAS status; in these cases outcome results in different treatment arms could be unpredictably affected by the unbalanced presence of NRAS mutations. Another heterogeneity element of these trials as well as of the present work is the associated chemotherapy doublet; in fact, we cannot exclude that differences in efficacy could be related to different schedule sequences (FOLFIRI vs. FOLFOX).

Our meta-analysis shows two elements: (1) a new descriptive analysis, with the purpose to quantify the benefit of a specific therapeutic strategy in mCRC RAS wt population. In fact, we collected predominantly data on “extended RAS” and mature follow-up informations, giving a direct and updated confirmation of data obtained by previous meta-analyses with different designs, (2) a critical perspective, discussing the limits of the present study and previous ones that infer on survival. The last element is fundamental: Overall Survival is a “synthetic” end-point influenced by all therapeutic lines. In our opinion, albeit the statements given by the present and previous works, the low rate of biologic cross-over (first anti-EGFR then bevacizumab or viceversa) (Table 1), due to a different plethora of reasons, could influence the clarity and the accuracy regarding the choice of biologic drug in the first line as best beneficial treatment. The question of which biologic drug to use and in what sequence to administer will remain open and controversial untill we design the “ideal” study described in Figure 1C. This is not only a methodological issue but also a pragmatic one.

Although some described limitations, this meta-analysis gives more robust information than a single trial. Randomized cross-over trials along with molecular characterization of patients are needed in order to provide more reliable data on the effect of starting therapy with different biologic drugs (anti-EGFR vs. bevacizumab) in mCRC. To date this question still remains open.

## Author contributions

AO and AD: contributed in planning, analysis, discussing, and writing of the manuscript; AN and MoC: contributed in planning and analysis of the manuscript; CD, CR, LS, AC, RC, ST: contributed in planning and writing of the manuscript; MiC, MB, GN, and AA: the author contributed in planning, writing, and discussing of the manuscript.

### Conflict of interest statement

The authors declare that the research was conducted in the absence of any commercial or financial relationships that could be construed as a potential conflict of interest.
